# Supplementary material for: Temporal analysis of two inducible human genes reveals post-transcriptional H3K4me3 deposition
Source: Life Sci Alliance. 2026 Apr 30;9(7):e202503511. doi: 10.26508/lsa.202503511 (PMC13135274; doi:10.26508/lsa.202503511)
Supplement: Supplementary file 1 [file LSA-2025-03511_SdataF4.docx]

**Source data file**


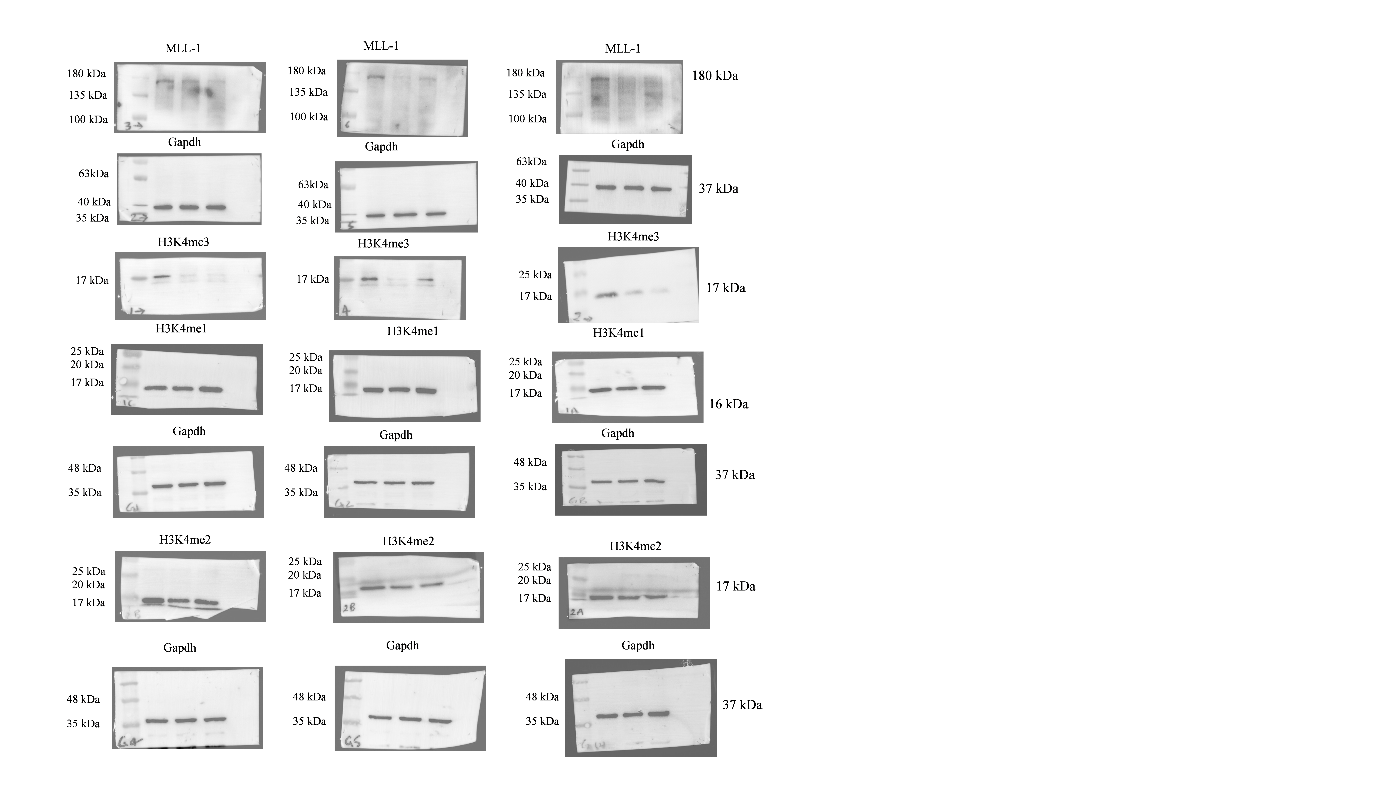


**Source data file: Uncropped Western blot images.** Western blot images showing protein levels of MLL1, H3K4me1, H3K4me2, H3K4me3, and GAPDH in THP-1 cells transduced with shMLL1 (n=2). Western was performed independently three times (n=3). MLL1 knockdown efficiency was validated by reduced MLL1 protein levels and corresponding decrease in H3K4me3, a downstream histone methylation mark. GAPDH was used as a loading control. These blots correspond to data presented in Fig 4A.
