# Supplementary material for: Temporal analysis of two inducible human genes reveals post-transcriptional H3K4me3 deposition
Source: Life Sci Alliance. 2026 Apr 30;9(7):e202503511. doi: 10.26508/lsa.202503511 (PMC13135274; doi:10.26508/lsa.202503511)
Supplement: Supplementary file 2 [file LSA-2025-03511_TableS1.docx]

**Supplementary Table S1**

**iNOS**

| *shMLL1# 1* | 5'-  CCGGGAGCTGTAAACATTCACATCTTCG  CAGAAGATGTAGGATTTAACAGTGCTTTTTG-3' | 5'-AATTCAAAAAGCACTGTTAAATCCTACATCTTCTGCGAAGATGTGAATGTTTACAGCTGCC-3' |
| --- | --- | --- |
| *shMLL1# 2* | 5'-CCGGCGCGCGTGATTACTCAATTTAACTCG  GATAATAATGGAATACGGAATACCGGCTTTTG  -3' | 5'- AATTCAAAAAGCCGGTATTTCCGTATTCCATTATTATCCGAGTTAAATTGAGTAATCACGCGCG-3' |

**Table S1**: shRNA oligonucleotide sequences used for human *MLL1* knockdown.
