# Supplementary material for: Temporal analysis of two inducible human genes reveals post-transcriptional H3K4me3 deposition
Source: Life Sci Alliance. 2026 Apr 30;9(7):e202503511. doi: 10.26508/lsa.202503511 (PMC13135274; doi:10.26508/lsa.202503511)
Supplement: Supplementary file 3 [file LSA-2025-03511_TableS2.docx]

**Supplementary Table S2**

**iNOS**

| **Gene** | **Forward primer sequence** | **Reverse primer sequence** |
| --- | --- | --- |
| *β2M* | 5'- AGGCTATCCAGCGTACTCCA -3' | 5'- CGGATGGATGAAACCCAGACA -3' |
| *TNF-α* | 5'- CTCTTCTGCCTGCTGCACTTTG -3' | 5'- ATGGGCTACAGGCTTGTCACTC -3' |
| *IL-1β* | 5'- CCACAGACCTTCCAGGAGAA -3' | 5'- GTGCAGTTCAGTGATCGTACAGG -3' |
| *MYC* | 5'- CCTGGTGCTCCATGAGGAGAC -3' | 5'- CAGACTCTGACCTTTTGCCAGG -3' |
| *TNF-α* promoter  (ChIP) | 5'- ATCAGTCAGTGGCCCAGAAG -3' | 5'- TCATCTGGAGGAAGCGGTAG -3' |
| *IL-1β* promoter  (ChIP) | 5'- ACCTTGGGTGCTGTTCTCTG -3' | 5'- CTGGTCTTGCAGGGTTGTGT -3' |
| *TNF-α* Exon 4  (ChIP) | 5'- TCTCCTACCAGACCAAGGTC -3' | 5'- CAAAGTAGACCTGCCCAGAC -3' |
| *IL-1β Intron*  *(ChIP)* | 5'- TAGAACACTGCAGCGCGAAC -3' | 5'- GCAACCTTCAATCCTGCTGC -3' |
| *GAPDH* Promoter  (ChIP) | 5'- TCATCCAAGCGTGTAAGGGT -3' | 5'- ACTGAGATTGGCCCGATGG -3' |
| *MYC* promoter  (ChIP) | 5'- GGGACTTCTTGATCAAAGCGC-3' | 5'- CGCATCCTTGTCCTGTGAGT-3' |
| *MyoD* promoter | 5'- CAATGCCTTGCCTCTCTCCA3' | 5'- CAAACCTCTCCAACACCCGA-3' |

**Table S2**. Oligonucleotide sequences used in ChIP-qPCR and RT-qPCR analysis.
