# Supplementary material for: Temporal analysis of two inducible human genes reveals post-transcriptional H3K4me3 deposition
Source: Life Sci Alliance. 2026 Apr 30;9(7):e202503511. doi: 10.26508/lsa.202503511 (PMC13135274; doi:10.26508/lsa.202503511)
Supplement: Supplementary file 4 [file LSA-2025-03511_TableS3.docx]

**Supplementary Table S3**

**iNOS**

| **S. No.** | | **Target** | **Application** | **Company** | **Catalogue No.** | | **Dilution (WB)** | **Amount (ChIP)** |
| --- | --- | --- | --- | --- | --- | --- | --- | --- |
| 1 | | GAPDH | Western | CST | 5174 | | 1:1000 | — |
| 2 | | H3K4me1 | Western/ChIP | Puregene | 73303 | | 1:2000 | 1.5 µg/IP |
| 3 | | H3K4me2 | Western/ChIP | Millipore | 07-030 | | 1:2000 | 1.5 µg/IP |
| 4 | | H3K4me3 | Western/ChIP | CST | 9751 | | 1:2000 | 1.5 µg/IP |
| 5 | | H3K27ac | ChIP | CST | 8173 | | — | 1.5 µg/IP |
| 6 | | H4K8ac | ChIP | Millipore | 07-328 | | — | 1.5 µg/IP |
| 7 | | Phospho-Rpb1 CTD (Ser2) | ChIP | CST | 13499 | | — | 1.5 µg/IP |
| 8 | | Anti-RNA polymerase II CTD repeat YSPTSPS (phospho S5) | ChIP | Abcam | Ab5408 | | — | 1.5 µg/IP |
| 9 | | NF-κB | ChIP | CST | 3033 | | — | 1.5 µg/IP |
| 10 | | MLL-1 | Western/ChIP | CST | 34907 | | 1:1000 | 1.5 µg/IP |
| 11 | | P300 | ChIP | CST | 54062 | | — | 1.5 µg/IP |
| 12 | | IgG | ChIP | CST | 2729 | | — | 1.5 µg/IP |
| 13 | Peroxidase AffiniPure® Goat Anti-Rabbit IgG | | Western | Jackson Immuno Research Laboratories | AB_2313567 | 1:10000 | | - |

**Table S3**. List of antibodies used in this study.
